# Knowledge of cervical cancer, human papillomavirus and prevention among first-year female students in residences at the University of the Free State

**DOI:** 10.4102/phcfm.v10i1.1637

**Published:** 2018-05-24

**Authors:** Nathaniel Mofolo, Maarasi Sello, Moleboheng Leselo, Naledi Chabanku, Samke Ndlovu, Quandry Naidoo, Gina Joubert

**Affiliations:** 1 Department of Family Medicine, University of the Free State, South Africa; 2 School of Medicine, University of the Free State, South Africa; 3 Department of Biostatistics, University of the Free State, South Africa

## Abstract

**Background:**

Cervical cancer is the second most common cancer among women in South Africa. One of the major risk factors for the development of cervical cancer is the human papillomavirus (HPV).

**Aim:**

To determine the knowledge of first-year female students living in residences on the main campus of the University of the Free State (UFS) regarding cervical cancer and HPV.

**Setting:**

Female residences on the main campus of UFS.

**Methods:**

A descriptive cross-sectional study was conducted on participants between the ages of 18 and 25 years using a non-random convenience sampling method. Seven residences were included. Anonymous self-administered questionnaires were distributed during the evening meetings to all first-year female students at the selected residences after an information session. Students had to complete the questionnaires immediately after the meeting.

**Results:**

Most of the 373 respondents (85.8%) knew that cervical cancer arises from the cervix, but only 15.4% knew that it was caused by a virus. Of the 62.5% participants who knew that HPV was a cancer-causing virus, most correctly knew that HPV was contracted by unprotected sexual intercourse (81.1%) and that there is a vaccine to protect against HPV (73.1%). However, 62.0% knew that the vaccine was available in South Africa and only 31.0% knew the vaccine was free of charge.

**Conclusion:**

The study revealed that students had limited knowledge of cervical cancer, HPV and vaccine availability.

## Introduction

Cervical cancer is one of the leading causes of death among women in South Africa.^1^ According to the 2017 summary report,^2^ 20.2 million South African women aged 15 years and older are at risk of cervical cancer. Each year, more than 7700 women are diagnosed with this disease and annually more than 4000 women die of cervical cancer in South Africa.^2^ Most of the deaths are attributed to late presentations at an advanced stage and the high prevalence of HIV because of its immune-compromising effect.

One of the major risk factors for the development of cervical cancer is the human papillomavirus (HPV). Other risks factors include immunosuppressive infections, long-term oral contraceptives and multiple pregnancies.^3^ Human papillomavirus is sexually transmitted and infects basal epithelial cells where it remains in a latent state.^4^ Some types of HPV cause abnormal cell changes that can eventually develop into cervical cancer.^4^ Infection with HPV is preventable; however, a large part of precaution depends on women’s knowledge and awareness.^3,5^

Cervical cancer can be prevented through primary and secondary preventative measures.^2,6^ Primary prevention involves targeting people who have not contracted HPV. This is achievable through behaviour modification to manage risk factors by introducing the idea of abstinence, mutual monogamy or using condoms.^7^ There are two effective vaccines, Cervarix^®^ and Gardasil^®^, available in South Africa for preventing HPV infections.^7^ In 2014, the HPV programme was launched by the Department of Health in conjunction with the Department of Education to vaccinate Grade 4 girls before they are exposed to HPV.^8^ Vaccination can protect men as well as women from genital warts linked to HPV. The targeted populations for vaccinations are preteens or individuals who are not yet engaging in sexual activities.^2^ The Centers for Disease Control and Prevention recommends that girls and boys of ages 11 or 12 years should be vaccinated; however, vaccinations can still be administered up to age 26 years, if not given previously.^9^

Secondary prevention focuses on screening and early detection, followed by treatment of precancerous lesions.^10^ Moss et al.^11^ reported that over half of new cervical cancer cases can be attributed to lack of screening. In 2010, the international recommendation was to do the first Pap smear at 21 years of age or within 3 years of sexual activity.^12^ The national policy on cervical cancer screening in South Africa allows women to get three Pap smears in a lifetime, taken every 10 years from the age of 30 years.^12^

Knowledge about cervical cancer and HPV is important for health promotion and prevention strategies. Studies^3,5,6,13^ have shown that young women have inadequate knowledge of cervical cancer and its link to HPV, and that there are misconceptions about their own susceptibility to HPV contraction.

### Aim

To determine the knowledge of first-year female students living in residences on the main campus of the University of the Free State (UFS) regarding cervical cancer and HPV.

## Research methods and design

### Study design, sample population and strategy

A descriptive cross-sectional study was conducted on first-year female students 18–25 years of age, living in residences on the Bloemfontein campus of UFS during February 2017. At the time of the study, there were 810 first-year female students in the 11 female-only residences. Seven residences were included in the study using convenience sampling.

### Data collection

Data were collected using a self-administered, anonymous questionnaire. To maintain anonymity, only the age of each participant had to be recorded. The questionnaire was designed by the researchers based on a questionnaire described in the literature^3^ as well as other subject-related information from the literature. The study questionnaire was only available in English.

To test the participants’ knowledge of cervical cancer, questions on causes, risk factors, methods of detection and possible methods of prevention were included. Questions to test the knowledge of HPV included how the virus could be contracted and whether it could cause cervical cancer. Questions on the HPV vaccine, such as availability, cost and level of protection against cervical cancer, were also included. Questions were structured as multiple choices, ‘Yes’, ‘No’ or ‘I don’t know’, or ‘True’ or ‘False’.

Questionnaires were distributed during the evening meetings to all first-year female students at the selected residences after an information session. Students had to complete the questionnaires immediately after the meeting. Completion and handing in of the questionnaire was considered as informed consent. Questionnaires were collected in a box after completion.

### Pilot study

The pilot study was conducted on 10 second-year medical students of the School of Medicine, UFS, using a non-random convenience sample. The participants completed the questionnaire and were asked to comment on how understandable and user-friendly the questionnaire was. The pilot study proved the questionnaire to be effective, easy to understand and that it took 10 min to complete. No changes were made to the questionnaire.

### Data analysis

Data were analysed by the Department of Biostatistics, Faculty of Health Sciences, UFS, using SAS version 9.3. Results were summarised by frequencies and percentages.

### Ethical considerations

The protocol was approved by the Health Sciences Research Ethics Committee, UFS (HSREC-S-30/2016). Permission to conduct the research on the campus was obtained from the Dean of Student Affairs and the Vice Rector Research. Participation in the study was voluntary.

## Results

All 432 questionnaires distributed were collected from the participants, and 373 (86.3%) were used in the study. Questionnaires of participants under the age of 18 years (*n* = 57) were excluded, one participant did not fill in her age and one did not complete any of the questions. The participants’ ages ranged between 18 and 25 years: 64.3% were 18 years of age, 22.5% were 19 years and 13.1% were between 20 and 25 years.

Table 1 indicates the percentages of participants who chose the correct options to the knowledge questions regarding cervical cancer, and the percentages who responded that they did not know. Most participants (85.8%) knew that cervical cancer arises from the cervix, but only 15.4% knew that it was caused by a virus. Almost half of the participants correctly noted that women who do not use protection (43.0%) and sexually active women (47.3%) were at risk to contract cervical cancer. Less than 20% knew that HIV-positive women (17.6%) and women on contraceptives (14.1%) are also more likely to contract cervical cancer.

**TABLE 1:** First-year female resident students’ knowledge of cervical cancer at the University of the Free State.

Questions on cervical cancer†	*n* (%)
**What is cervical cancer? (*n* = 372)**	
A cancer arising from the cervix	319 (85.8)
I don’t know	10 (2.7)
**What causes cervical cancer? (*n* = 319)**	
A virus	49 (15.4)
I don’t know	88 (27.6)
**Who is more likely to contract cervical cancer? (*n* = 319)‡**	
HIV-positive woman	56 (17.6)
Women who don’t use protection	137 (43.0)
Sexually active women	151 (47.3)
Women on contraceptives	45 (14.1)
I don’t know	62 (19.4)
**Cervical cancer can be detected by: (*n* = 319)**	
Pap smear	191 (59.9)
I don’t know	42 (13.2)
**Cervical cancer can kill you (*n* = 319)**	
True	272 (85.3)
**You cannot control the development of cervical cancer because all women eventually get it (*n* = 319)**	
False	291 (91.2)
**Teenagers who are sexually active from a young age are at risk of developing cervical cancer (*n* = 319)**	
True	264 (82.8)
**There are vaccines against cervical cancer (*n* = 319)**	
True	178 (55.8)
**A Pap smear is a test used to detect cervical cancer (*n* = 319)**	
True	258 (80.9)
**A Pap smear is used to clean the womb (*n* = 319)**	
False	200 (62.7)

† , for each question only the correct response(s) and the ‘I don’t know’ response out of the available options in the questionnaire are listed.

‡ , more than one response correct.

*n*, number.

Most participants knew that cervical cancer can be fatal (85.3%), that the development of cervical cancer can be controlled (91.2%), that sexually active teenagers are at risk (82.8%) and that a Pap smear is a test to detect cervical cancer (80.9%). Only 55.8% knew that there are vaccines available against cervical cancer.

Most of the participants knew that sexually contracted diseases (78.2%) and multiple sexual partners (80.4%) were risk factors for cervical cancer (Table 2). Less than half of the participants (44.8%) knew that unhygienic toilet seats were a risk factor, while 25.2% knew that blood transfusions were a risk factor. Few participants correctly selected drinking dirty water (3.8%) and smoking cigarettes (12.6%) as risk factors. Almost 60% of the participants (57.4%) knew that drinking alcohol was not a risk factor.

**TABLE 2:** First-year female resident students’ knowledge of the risk factors and preventative measures of cervical cancer at the University of the Free State.^13,14,15^

Questions on cervical cancer†	Answered correctly	I don’t know
	*n* (%)	*n* (%)
**What are the risk factors of cervical cancer? (*n* = 317)**		
Sexually contracted diseases (Yes)	248 (78.2)	45 (14.2)
Multiple sexual partners (Yes)	255 (80.4)	45 (14.2)
Unhygienic toilet seats (Yes)	142 (44.8)	76 (24.0)
Drinking dirty water (Yes)	12 (3.8)	124 (39.1)
Blood transfusions (Yes)	80 (25.2)	123 (38.8)
Drinking alcohol (No)	182 (57.4)	108 (34.1)
Smoking cigarettes (Yes)	40 (12.6)	107 (33.8)
**The following can help prevent cervical cancer: (*n* = 318)**		
Condoms (Yes)	185 (58.2)	79 (24.8)
HPV vaccine (Yes)	221 (69.5)	80 (25.2)
Contraceptive (No)	112 (35.2)	138 (43.4)

† , for each question only the correct responses out of the available options in the questionnaire are listed.

HPV, human papillomavirus; *n*, number.

A majority of participants (69.5%) knew that the HPV vaccine can help prevent cervical cancer compared to 58.2% who stated condoms as a preventative measure. Only 35.2% stated correctly that contraceptive does not help prevent cervical cancer.

Of the 319 participants who answered that cervical cancer arises from the cervix, 60.1% learned this while in school, 33.0% via the media, 10.4% from other sources, such as family, friends or physician, and 2.8% at UFS.

Table 3 indicates the percentages of participants who chose the correct options to the knowledge questions regarding HPV and the percentages who responded that they did not know. Of the 62.5% of participants who knew that HPV was a cancer-causing virus 81.1% correctly knew that HPV was contracted by unprotected sexual intercourse and 73.4% knew that there is a vaccine to protect against HPV. However, 62.0% knew that the vaccine was available in South Africa and only 31.0% knew the vaccine was free of charge.

**TABLE 3:** First-year female resident students’ knowledge of the human papillomavirus at the University of the Free State.

Questions on HPV†	*n* (%)
**What is human papillomavirus (HPV)? (*n* = 373)**	
A virus that causes cancer	233 (62.5)
I don’t know	128 (34.3)
**How is HPV contracted? (*n* = 233)**	
Unprotected sexual intercourse	189 (81.1)
I don’t know	28 (12.0)
**Is there a vaccine for protection against HPV? (*n* = 233)**	
Yes	171 (73.4)
I don’t know	4 (1.7)
**Is the HPV vaccine available in South Africa? (*n* = 171)‡**	
Yes	106 (62.0)
I don’t know	61 (35.7)
**Is the HPV vaccine free of charge? (*n* = 171)‡**	
Yes	53 (31.0)
I don’t know	71 (41.5)
**HPV is a sexually transmitted infection (*n* = 233)**	
True	155 (66.5)
**You can have HPV for many years and not show symptoms (*n* = 233)**	
True	197 (84.6)

† , for each question only the correct response out of the available options in the questionnaire is listed.

‡ , for participants who have heard of the HPV vaccine.

HPV, human papillomavirus; *n*, number.

In total, 200 participants knew what cervical cancer and HPV are. Of these, 71.0% knew that HPV causes cervical cancer, while 24.5% did not know. Similarly, 71.5% knew that the HPV vaccine did not 100% protect women against cervical cancer, while 23.5% did not know.

Of the 190 participants who answered the last section of the questionnaire, 136 (71.6%) did not believe they are at risk of developing cervical cancer, while 152 (81.3%) out of 187 participants did not believe they are at risk of contracting HPV.

## Discussion

In a study conducted in 1997,^16^ it was found that rates of HPV infection are the highest in adults between the ages of 18 and 28 years. This was the rationale for targeting study participants aged 18–25 years. The summary report by Bruni et al.^2^ revealed that the burden of HPV infection is affecting younger females from an early age, and makes cervical cancer the most common cancer among South African women between the ages of 15 and 44 years.

Most of the participants (85.8%) were aware of what cervical cancer is. This is lower than the 95% found in a study performed at the University of the Witwatersrand,^6^ but higher than the 42.9% found at a university in Kwazulu-Natal.^17^ The main sources of knowledge reported by the participants were school (60.1%) and the media (33.0%). Media plays an important role in getting information to the youth, which can create and raise awareness about cervical cancer and HPV. The sexual development of teenagers is one of the most important journeys into adulthood, and can easily be influenced by media messages on sex and sexuality.^18^ None of the participants indicated that they received information from their health practitioners. With increasing prevalence of cervical cancer in South Africa, health practitioners can play a major role in health promotion and prevention. The more knowledgeable the public, the better choices they can make towards their own health.

According to the World Health Organization, approximately 30%–60% of all sexually active adults may contract HPV during their lifetime. In addition, young females are at risk as they are more inclined to be sexually active and have more sexual partners.^19^ Even though 85.8% of participants were aware of cervical cancer, almost 20% less were aware of what HPV is (62.5%), showing that vast effort needs to be placed in educating women on oncogenic HPV (strains 16 and 18).^15^

In this study, the participants correctly identified that having multiple sexual partners (80.4%) is a risk factor for developing cervical cancer. This is higher than the 52% reported by Kulua^6^ and 31.1% reported by Hoque et al.^17^ Pitts and Clarke^13^ reported an overall low awareness of risk factors for cervical cancer among those knowledgeable of HPV infection.

Almost 81% of the participants selected Pap smear test as a method of detection of cervical cancer. Results from the Witwatersrand study found that 82% of the participants were aware of the test, but only 60% were aware that it was used for cervical cancer detection.^6^ Knowledge about the indication of a Pap smear is important for early detection of cervical cancer or its pre-neoplastic lesions to enable easier intervention.

Most (73.4%) of the participants who had heard about HPV were aware that a vaccine is available, but only 62.0% of these participants knew that the vaccine was available in South Africa. This shows a need for education about the vaccine to empower young women to protect themselves from contracting HPV.

### Study limitations

The study has several limitations, suggesting that the results should be interpreted with some caution. The study used convenience sampling, which may result in selection bias. Another potential limitation of this study is the reliance on self-reported data. Therefore, responses to sensitive questions may be biased.

Some of the participants appeared to be helping each other in answering some of the questions and some questionnaires were incomplete. A number of participants did not answer the last few questions, as they did not see these on the back page of the questionnaire.

The fact that the study did not record the faculty where the participants study means that we cannot ascertain whether the overrepresentation of a faculty, such as Health Sciences, may have affected the results, nor can it be ascertained whether certain faculties’ students are less knowledgeable, which makes targeted education impossible.

## Conclusion

The study is consistent with other studies performed at universities in South Africa and internationally, which shows that female students have limited knowledge of cervical cancer, HPV and the important link between the two.

In conclusion, there is an urgent need for provision of sexual and women’s reproductive health programmes on campus. The data from this study suggest that, in order to enhance knowledge about cervical cancer and HPV immunisation uptake, provision of information about HPV infection and its link to cervical cancer and risk factors is essential, with emphasis on high HPV infection rates, provision of assurance on safety and efficacy of the new vaccine and eliminating stigma associated with the prevention of sexually transmitted infections. Education on the prevention of cervical cancer needs to start at primary school level, where vaccination can be done from the age of 9 years. This needs to continue up to tertiary level, while also taking care of other preventative strategies such as abstinence, monogamous sexual relations and condom use.

### Recommendations

Education about HPV and its relationship to cervical cancer can be included in university programmes such as UFS101, a core compulsory module for all first-year students at UFS, and in life orientation in high schools.

1. Education must include the use of condoms to prevent HPV and other sexually transmitted diseases.
2. Student health clinics and community organisations should raise awareness about cervical cancer, its prevention and early detection.
